# Venous malformations: PIK3CA mutations guide new treatments

**DOI:** 10.18632/oncotarget.9252

**Published:** 2016-05-09

**Authors:** Hashem A. Dbouk

**Affiliations:** Department of Pharmacology, University of Texas Southwestern Medical Center, Dallas, TX, USA

**Keywords:** PIK3CA, PI3K, vascular, venous malformation

The Phosphoinositide 3-Kinase (PI3K) pathway has been extensively studied in tumors due its roles in promoting cellular growth and proliferation [1]. The most common PI3K mutations are in the *PIK3CA* gene encoding the p110α catalytic subunit, including the “hotspot” activating mutations E545K and H1047R that can lead to constitutive signaling of the pathway [1]. Consequently, activation of the serine/threonine kinase Akt can promote proliferative and cell growth pathways through regulation of mTOR and other intermediates [1]. In addition to driving tumorigenesis, hotspot *PIK3CA* mutations have also been shown to drive a wide spectrum of non-malignant over-growth disorders collectively termed the *PIK3CA*-Related Overgrowth Spectrum [2]. More recently, mutations in *PIK3CA* have been identified in venous malformations (VMs) [3], the most frequent form of vascular malformations with a frequency of about 1 in 5000 people in the general population [4]. These painful and often disfiguring lesions are characterized by endothelial cell overgrowth, loss of supporting mural cells, and a disorganized extracellular matrix resulting in dilated and distended vessels in a variety of tissues, with common occurrence in the cutaneous layer of the skin [4].

Both ubiquitous and endothelial-specific *PIK3CA* knockout mouse models display lethality due to angiogenic defects, directly implicating p110α in vasculogenesis [5]. Prior to the identification of *PIK3CA* mutations in VMs, the only known mutations were in the *TEK* gene that encodes the endothelial-specific Tie2 RTK. Mutations of *TEK* cause ligand-independent autophosphorylation of the receptor and drive signaling primarily to the PI3K and the MAPK pathways [4]. Tie2 mutations occur in about 50% of VM patients while *PIK3CA* mutations account for about 25% of patients with apparent mutual exclusivity [3], suggesting a functional redundancy between the two disease drivers. Two recent studies confirm the presence of *PIK3CA* mutations in VMs from human patients and support the notion that hyperactivation of the Tie2-PIK3CA-Akt pathway is responsible for lesion presentation [6, 7]. Castel *et al* used two mouse models expressing the H1047R *PIK3CA* mutant in either an endothelial compartment or sporadically in the body to show that aberrant PI3K signaling results in the development of VMs [6]. Similarly, Castillo *et al.* expressed H1047R *PIK3CA* in the embryonic mesoderm in a mosaic fashion and in a heterozygous state, analogous to human disease context, and observed the development of subcutaneous VMs in different body sites [7]. It is interesting to point out that in both studies, while the expression of the oncogenic H1047R *PIK3CA* mutant occurred sporadically in different tissues, the overwhelming phenotype observed was the appearance of VMs, suggesting cells of origin are more susceptible to PI3K perturbations compared to other cells in the body [6, 7].

The implication of the PIK3CA-Akt-mTOR pathway as the driver behind VMs may be grounds to use drugs that target this pathway for treatment of VMs. Current treatment options for VMs are sclerotherapy or surgical removal, both of which are limited by anatomic location and suffer from the risk of lesion recurrence [4]. Intriguingly, a recent pilot study has demonstrated the efficacy of Rapamycin in blocking VM-associated phenotypes, including endothelial cell proliferation and pericyte coverage of endothelium, ultimately leading to regression of VMs in a cohort of patients [3, 6, 7]. Similarly, the use of the p110α-specific inhibitor, BYL719, showed a marked decrease in VM size, blocked endothelial cell proliferation, and unlike Rapamycin induced apoptosis of the expanded endothelial cells [3, 6]. The use of BYL719 may provide an advantage over the use of Rapamycin by avoiding the immune-suppressive effects of the mTOR inhibitor while also targeting a node in the signaling cascade upstream of mTOR, thereby mitigating hyperactivation of the PI3K-Akt pathway downstream of Tie2. Castel *et al.* also used the BYL719 in two different cream formulations that allow topical administration of the PI3K inhibitor for treatment of cutaneous VM lesions, potentially abolishing the need for systemic treatments that increase toxicity and off-target effects [6]. Notably, the use of either Rapamycin or BYL719 was shown to be effective against VMs driven by both Tie2 and PIK3CA mutations [3, 6].

These exciting studies identify the effects sporadic PIK3CA activating mutations can have in driving VMs [3, 6, 7], adding to the list of *PIK3CA*-Related Overgrowth Spectrum diseases. Importantly, these studies provide support for exploring *PIK3CA*-targeted therapeutics that can block and reverse the development of VMs, potentially improving the quality of life for patients afflicted by these lesions. Equally important, these results also raise the question of why endothelial cells are more sensitive to *PIK3CA* mutations compared to other cells in the body. Furthermore, while *TEK* and *PIK3CA* mutations cause generally similar VM phenotypes in mice, analysis of patients reveals potential differences in the characteristics of the developing VM lesions that suggest different cells of origin and other factors which may affect the development of these vascular overgrowths [3]. Significantly, VMs from patients that do not carry mutations in *TEK* or *PIK3CA* represent highly distinct characteristics [3], pointing towards additional pathways that may drive VM development and highlighting the need for further research.
